# An interpretable machine learning model for predicting prognosis in acute ischemic stroke with large vessel occlusion

**DOI:** 10.3389/fneur.2026.1842324

**Published:** 2026-09-09

**Authors:** Meng Li, Jia Wei, Guangshun Lu, Hongjuan Zhao, Li Meng, Haihua Bao

**Affiliations:** Department of Medical Imaging Center, Affiliated Hospital of Qinghai University, Xining, China

**Keywords:** Acute ischemic stroke, collateral status, large-vessel occlusion, multiphase CT angiography, perfusion imaging

## Abstract

**Objective:**

To develop and validate an interpretable nomogram that integrates an imaging score, clinical characteristics, and machine-learning models to predict 90-day functional outcomes in patients with acute ischemic stroke (AIS) and large-vessel occlusion (LVO).

**Methods:**

We analyzed 240 AIS patients with anterior circulation LVO who underwent one-stop multimodal CT between October 2019 and December 2024. Fifty-two variables, including pretreatment clinical features, conventional and advanced imaging, and angiographic characteristics, were assessed. The 90-day modified Rankin Scale (mRS-90) was used as the prognostic endpoint; good and poor outcomes were defined as mRS-90 ≤ 2 and > 2, respectively. Patients were randomly assigned to training (80%, *n* = 192) and testing (20%, *n* = 48) cohorts. Least absolute shrinkage and selection operator (LASSO) regression was used to derive the imaging score, which was then combined with key clinical predictors to construct a nomogram. Clinical, imaging, and hybrid models were developed separately and evaluated using the area under the receiver operating characteristic curve (AUC), calibration curves, and decision curve analysis (DCA). Repeated stratified 5-fold cross-validation repeated 20 times was performed as a stability analysis of the fixed final model structures. SHapley Additive Explanations (SHAP) was applied to identify influential features and visualize feature importance and interactions.

**Results:**

Among 240 patients, 162 (67.5%) had unfavorable 90-day functional outcomes. Patients with unfavorable outcomes were generally older, had higher admission NIHSS scores, and showed less favorable collateral and perfusion profiles. Age, admission NIHSS score, and onset-to-CT time were retained as clinical predictors, while eight imaging features were integrated into the imaging score. Adding the imaging score did not significantly improve discrimination in the testing cohort (ΔAUC, 0.0078; 95% CI, −0.0071 to 0.0227; *P* = 0.305). In repeated stratified 5-fold cross-validation performed 20 times, the mean AUCs were 0.942, 0.747, and 0.939 for the clinical, imaging, and hybrid models, respectively. SHAP analysis characterized the contributions of age, NIHSS, onset-to-CT time, and the composite imaging score within the four-input hybrid model.

**Conclusions:**

An interpretable model combining routine clinical predictors with a multimodal CT-derived imaging score demonstrates promising predictive potential for 90-day functional outcome in patients with AIS-LVO.

## Introduction

Stroke remains the second leading cause of death and the third leading cause of disability related deaths worldwide, with increasing evidence indicating that the burden of stroke is increasing throughout the life cycle, and the incidence rate of stroke among young people is also rising (1, 2). The prevalence of stroke among people under 70 years old is up 22.0%, and the incidence rate is up 15.0%. Anterior circulation blockage accounts for about 70%-80% of AIS-LVO cases.

Mechanical thrombectomy (MT) is the standard treatment for acute ischemic stroke caused by LVO (3). Although MT benefits many patients with LVO stroke, previous studies indicate that nearly half remain dependent on others for daily care at 90 days, and 90-day mortality remains substantial (4). Another study showed that, during 3–6 months of follow-up after onset, patients with LVO had more than twice the risk of functional dependence [modified Rankin Scale (mRS) score ≥ 3] or death compared with patients without LVO (5). Therefore, establishing a reliable prognostic assessment system for patients with LVO stroke has important clinical value. Preoperative prediction of unfavorable outcomes is particularly important because it can optimize individualized treatment decisions and reduce social and economic burdens by avoiding ineffective treatment.

Current patient screening relies mainly on clinical assessment and imaging evaluation. Clinical assessment includes symptom severity and baseline functional status, whereas imaging evaluation estimates the extent of irreversible brain injury. Patients with large infarct cores are less likely to benefit from thrombectomy and may have a higher risk of hemorrhage. Imaging techniques have therefore been increasingly used to predict ischemic stroke prognosis (6, 7). For high-risk patients, early identification may support timely transition to alternative treatment strategies, improve the efficiency of medical-resource utilization, and enhance quality of life. However, the long acquisition time and high cost of MRI limit its widespread use in emergency settings.

Multimodal CT can support practical decision-making in emergency stroke care and may be associated with final functional outcomes (8, 9). A typical multimodal CT protocol for stroke includes non-contrast CT (NCCT), CT perfusion (CTP), and CT angiography (CTA), which can usually be completed within 10 min. In AIS, collateral circulation is a key determinant of infarct volume, endovascular treatment response, and clinical prognosis (10). Effective collateral circulation can extend the treatment window after stroke and increase the brain's tolerance to ischemia and hypoxia. ColorViz fusion images can shorten the time required for vascular grading and may improve inter-rater consistency (11). This technique evaluates collateral circulation, assesses perfusion status, and shows good correlation with digital subtraction angiography (DSA) collateral grading, thereby supporting endovascular-treatment selection (12). CTP is also essential for guiding intravenous thrombolysis and MT. Time to maximum of the residue function (Tmax) is a sensitive perfusion parameter used to identify hypoperfused tissue. Higher Tmax thresholds indicate more severe perfusion delay; in particular, Tmax > 6 s has been widely used to define severely hypoperfused tissue in AIS (13). However, multimodal CT generates large amounts of cross-modal imaging data. Clinicians must integrate complex clinical, laboratory, and imaging information within a short time, which increases both interpretive complexity and time costs. It remains unclear how treatment decisions based on this information can most effectively improve outcomes. A review of the existing literature suggests that few studies have evaluated machine-learning models for predicting short-, medium-, and long-term functional prognosis in patients with AIS caused by anterior-circulation artery occlusion. The relationships among CTP parameters, collateral grading, and prognosis in AIS-LVO remain uncertain. With the increasing use of perfusion imaging in patients presenting in the late time window, the prognostic value of one-stop multimodal CT combined with clinical information warrants further investigation.

The objective of this study was to develop machine-learning models using clinical, laboratory, and multimodal imaging data to predict 90-day functional outcomes in patients with AIS and anterior-circulation LVO.

## Materials and methods

### Data sources and study patients

This retrospective study received approval from the Ethics Committee of the Affiliated Hospital of Qinghai University. Given the retrospective nature of this investigation, the review board waived the requirement for written informed consent. We analyzed 240 patients with clinically diagnosed AIS and anterior circulation large vessel occlusion who underwent one-stop imaging of stroke at the Medical Imaging Center of Qinghai University Affiliated Hospital from October 2019 to December 2024 for analysis To achieve a balance between images and data, all cases were randomly divided into a training cohort (*n* = 192) and a testing cohort (*n* = 48) in an 8:2 ratio.

Inclusion criteria encompassed:

(1) Acute anterior circulation ischemic stroke confirmed as unilateral by magnetic resonance imaging (MRI), with middle cerebral artery/internal carotid artery occlusion verified via CTA/DSA.
(2) Eligible patients were adults aged ≥ 18 years; (3) Underwent multimodal CT imaging comprising NCCT, CTA, and CTP.

Exclusion criteria comprised:

(1) Patients exhibiting motion artifacts or insufficient contrast agent administration.
(2) Incomplete CTA/CTP examinations.
(3) Incomplete clinical data.
(4) Presence of intracranial hemorrhage or multiple intracranial vascular occlusions.
(5) Suboptimal image quality precluding accurate assessment. (6) Suffering from severe heart, lung, kidney, liver or other major organ diseases.

### Clinical data acquisition

Clinical information was systematically extracted from electronic medical records documented by neurology specialists trained in stroke diagnosis and management during admission. Laboratory investigations utilized results obtained within 24 h post-admission. Radiological feature extraction was independently performed by two board-certified neuroradiologists with over five years' experience.

### Baseline clinical parameters collected

Demographic characteristics (age, sex, education level), cerebrovascular risk factors (hypertension, diabetes mellitus, hyperlipidemia, prior stroke history, atrial fibrillation history, coronary artery disease history, smoking status, alcohol consumption), time interval from symptom onset to initial imaging, National Institutes of Health Stroke Scale (NIHSS) score at admission, treatment modality, and modified Rankin Scale (mRS) scores at 3 months. The 90-day modified Rankin Scale score was used as the prognostic endpoint. Favorable outcome was defined as mRS ≤ 2 and coded as 1, whereas unfavorable outcome was defined as mRS > 2 and coded as 0 (14). Telephone follow-up was conducted for outcome verification. Laboratory parameters included total cholesterol (TC), triglycerides (TG), high-density lipoprotein cholesterol (HDL-C), low-density lipoprotein cholesterol (LDL-C), white blood cell count (WBC), lymphocyte count, neutrophil count, monocyte count, red blood cell count (RBC), hemoglobin (HGB), platelet count (PLT), mean platelet volume (MPV), homocysteine, creatinine (CR), and C-reactive protein (CRP).

### Data extraction and imaging: conventional imaging signs

#### Image acquisition and reconstruction

All patients underwent our institutional acute ischemic stroke imaging protocol using either a 128-slice spiral CT scanner (Discovery CT 750 HD, GE Healthcare, Milwaukee, WI, USA) or a 512-slice spiral CT scanner (Revolution CT, GE Healthcare, Milwaukee, WI, USA). The imaging protocol commenced with NCCT, followed by whole-brain perfusion CT acquisition in volumetric shuttle mode, spanning from the skull base to the vertex. Perfusion imaging comprised continuous acquisition over 28 rotations, completed within 48 s using a pitch of 0.984 mm and rotation time of 0.4 s. Subsequently, a 30 mL saline flush was administered at a constant flow rate. Raw data were reconstructed with 0.625 mm slice thickness.

mCTA was performed 5 min after the initial scan. The arterial phase employed automated bolus tracking to achieve peak enhancement, encompassing the neurovascular anatomy from the aortic arch to the vertex. Venous and late venous phases were limited to intracranial coverage. An 8-s interval was maintained between consecutive phases (arterial-to-venous and venous-to-late venous). Vascular information from all mCTA phases was processed into temporally resolved, color-coded maps using FastStroke software (GE Healthcare). ColorViz color coding reflected the temporal delay of collateral filling relative to the contralateral hemisphere: red indicated no or minimal delay, green a one-phase delay, and blue a delay of two or more phases.

#### Feature selection and data processing

All NCCT, CTP, and CTA images were independently evaluated by two board-certified neuroradiologists (with >5 years of dedicated neuroradiology experience), who correlated findings with each patient's comprehensive imaging and clinical dataset to confirm AIS diagnosis. Radiological characteristics documented included baseline ASPECTS score, presence of basal ganglia haziness sign, insular ribbon sign, occluded vessel location, occlusion segment, affected side, ColorViz score, and multiphase CTA collateral grading.

The Alberta Stroke Program Early CT Score (ASPECTS) was calculated on axial non-contrast NCCT reconstructions at 5 mm thickness. Occlusion sites were identified on axial maximum intensity projections (MIPs) derived from mCTA datasets.

A dual-parameter collateral circulation scoring system based on temporally resolved color maps employed axial color-coded MIPs to categorize both collateral filling extent and delay across three tiers (15): ColorViz collateral status was assessed on time-variant color-coded multiphase CTA maps. The score was defined according to the extent and temporal delay of collateral vessel opacification in the ischemic territory compared with the contralateral hemisphere. A score of 1 indicated normal or near-normal collateral filling with no obvious delay, predominantly shown as red vessels. A score of 2 indicated moderately delayed collateral filling, typically corresponding to a one-phase delay and predominantly green vessels. A score of 3 indicated severely delayed or absent collateral filling, corresponding to a two-phase delay or non-visualization of collateral vessels, predominantly blue or absent vessels. Lower scores represented better collateral circulation, whereas higher scores indicated poorer collateral status.

Collateral grading on mCTA utilized the 0–5 point scale proposed by Bijoy K. Menon et al. (16). Anterior circulation large vessel occlusion was defined as obstruction involving the internal carotid artery (intracranial segment), M1 segment, or proximal M2 branches of the middle cerebral artery (17). Moderate to good collateral status was defined as collateral scores ≥ 3 (18).

Additionally extracted parameters comprised: relative cerebral blood flow (rCBF) < 20%,rCBF < 30%, rCBF < 34%, rCBF < 38%; Tmax > 4s, Tmax > 6s, Tmax > 8s, Tmax > 10s; cerebral blood volume (CBV) < 34%, CBV < 38%, CBV < 42%, mismatch volume, mismatch ratio, and hypoperfusion index (HIR). Automated intelligent post-processing of CTP images was performed using the AI-powered imaging analytics software uAI Research Portal (Shanghai United Imaging Intelligence) (19). The HIR was defined as the volumetric ratio of tissue exhibiting Tmax > 10s vs. Tmax > 6s. The CBV index was calculated as the mean CBV within ipsilateral ‘Tmax > 6s lesions' divided by the mean CBV across the corresponding ipsilateral territory (20).

When discordance occurred between the two physicians regarding imaging findings, consultation was sought from a chief neuroradiologist with 20 years of diagnostic experience to achieve consensus. Missingness was assessed before model development. All 240 patients had complete clinical, laboratory, imaging, and 90-day outcome data. The training cohort was used for preprocessing, predictor selection, and model fitting, whereas the testing cohort was reserved for performance evaluation. Variables exhibiting *P*-values < 0.05 underwent collinearity assessment through calculation of variance inflation factors (VIF) and tolerance metrics. Absence of significant collinearity was defined by tolerance >0.1 or VIF < 5. Non-collinear variables were subsequently incorporated into the Least Absolute Shrinkage and Selection Operator (LASSO) regression model.

### Model construction

The dataset was randomly partitioned at an 8:2 ratio into training cohort and testing cohort to ensure independent model development and validation. Clinical feature selection commenced with univariate screening. Favorable 90-day functional outcome, defined as a modified Rankin Scale score of 0–2, was treated as the positive class. Continuous variables were compared between outcome groups using the independent-samples *t* test when normally distributed and the Mann–Whitney *U* test when the normality assumption was not satisfied. Categorical variables were compared using the chi-square test or Fisher's exact test, as appropriate. Variables meeting the prespecified univariate screening criterion were subsequently considered for multivariable logistic regression. Significant variables from univariate analysis subsequently underwent multivariate logistic regression with stepwise selection to identify independent predictive factors (*P* < 0.05). Imaging features selection employed LASSO regression for dimensionality reduction and feature selection. Optimal regularization parameter (λ) was determined through 10-fold cross-validation, retaining imaging features corresponding to non-zero coefficients at the selected λ value.

Three predictive models were constructed based on the selected features:

Clinical Model: Independent clinical predictors identified via multivariate logistic regression served as inputs for a logistic regression-based clinical prediction model.

Imaging Model: Imaging features selected by LASSO regression were used as inputs for a logistic regression-based imaging prediction model. Continuous predictors were z-score standardized using training-cohort means and standard deviations. Ordinal and binary variables retained their prespecified codes. Thus, for each continuous predictor, the standardized value was calculated as Z = (X –meantraining)/ SDtraining (Supplementary). Concurrently, an imaging score was calculated using the retained imaging features weighted by their respective LASSO regression coefficients, following the formula:


$$\begin{array}{l} \mathrm{Imaging}\;\mathrm{Score}=\mathrm{Intercept}+\Sigma \left(\mathrm{Coefficient}\;\times \;\mathrm{Variable}\right). \end{array}$$

Hybrid model: The combined model was a four-variable model incorporating age, NIHSS, onset-to-CT time, and Imaging score. Imaging score represented the weighted composite contribution of the eight imaging variables selected by LASSO. SHapley Additive Explanations (SHAP) were used to interpret the hybrid model incorporating age, NIHSS, onset-to-CT time, and the Imaging score. To improve clinical utility and interpretability, a multimodal CT-based nomogram was constructed incorporating the imaging score from the imaging model alongside independently significant clinical predictors, providing a visual decision-making tool.

### Model evaluation

Discrimination was quantified using the area under the receiver operating characteristic curve (AUC), with 95% confidence intervals estimated using the DeLong method. Because predictions from the clinical and hybrid models were obtained for the same patients, their AUCs were compared using the paired DeLong test. Accuracy, sensitivity, and specificity were calculated using a probability threshold of 0.50. Calibration curves were used to compare predicted and observed probabilities, and decision curve analysis was performed to evaluate net benefit across threshold probabilities.

To evaluate sensitivity to a single random data partition, repeated stratified 5-fold cross-validation was performed using the entire cohort, with 5-fold cross-validation repeated 20 times and outcome stratification maintained in each fold. The clinical, imaging, and hybrid logistic-regression model structures were refitted in the analysis folds and evaluated using out-of-fold predictions from the corresponding assessment folds. For each repetition, the five assessment folds were combined to calculate one AUC. Performance was summarized using the mean, standard deviation, median, and 2.5th−97.5th percentile interval across repetitions. The paired AUC difference between the hybrid and clinical models was calculated within each repetition.Model interpretability was enhanced using SHapley Additive Explanations (SHAP) analysis to quantify individual feature contributions to predictive outcomes. This approach provides global feature importance rankings and instance-specific explanations for individual predictions, thereby enhancing model transparency and clinical interpretability.

### Statistical analysis

Statistical analyses were performed using R software (version 4.6.1; https://www.r-project.org/.) and IBM SPSS Statistics (version 22.0; IBM Corporation, Armonk, NY, USA). Continuous variables are presented as mean ± standard deviation, with between-group comparisons conducted via *t*-test or Mann–Whitney *U* test where appropriate. Categorical variables are expressed as counts and percentages, analyzed using Fisher's exact test or chi-square test for group comparisons. The cohort was randomly partitioned at an 8:2 ratio into a training cohort (*n* = 192) and testing cohort (*n* = 48). Multivariate regression models were employed to identify independent predictors. A two-tailed *P*-value < 0.05 was considered statistically significant. The uAI Research Portal (19) was utilized for performing LASSO regression, generating receiver operating characteristic (ROC) curves, calibration plots, decision curve analysis (DCA), and conducting multivariate logistic regression analyses.

## Results

### Patient characteristics

**Figure 1:**
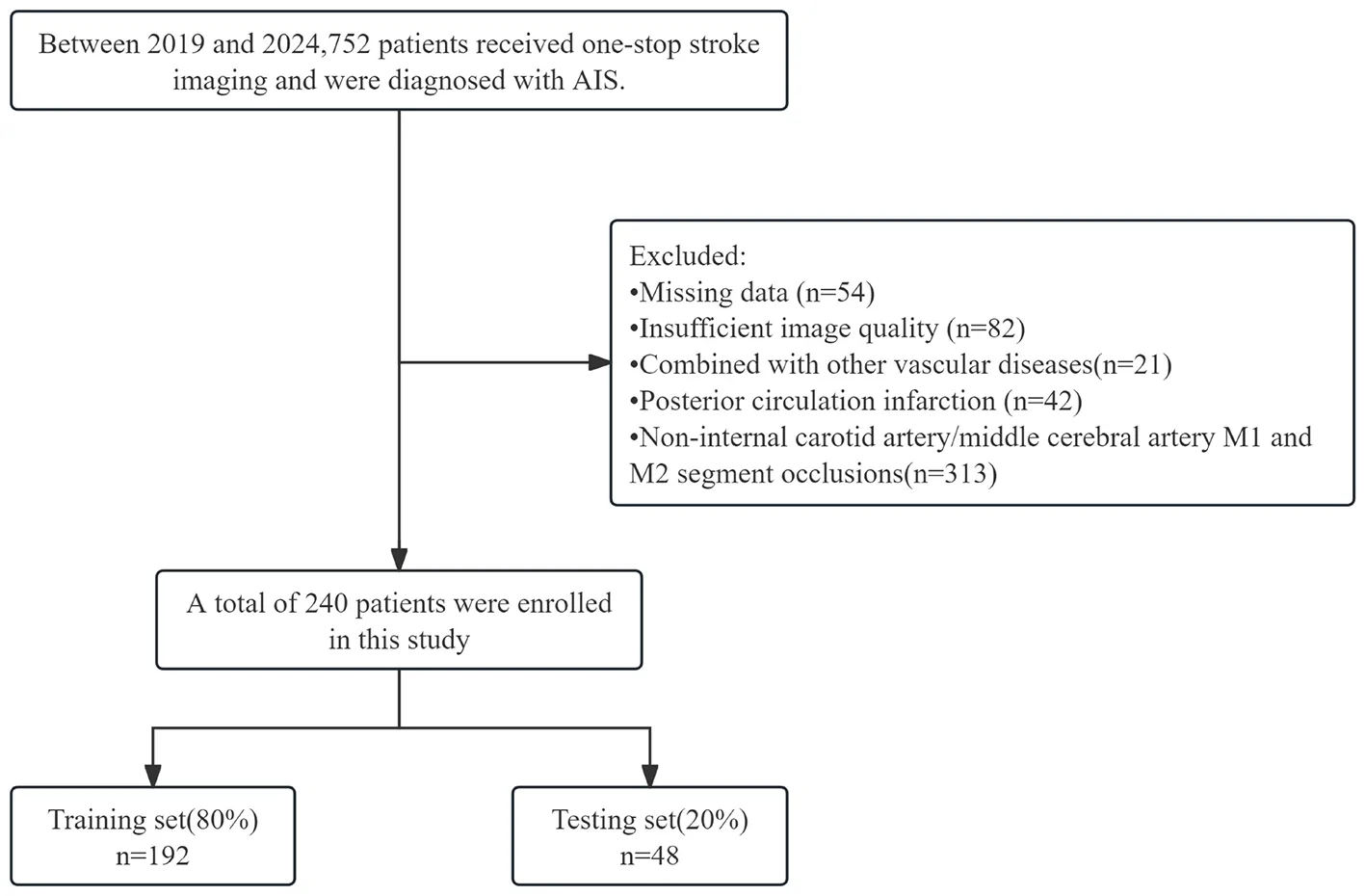
Patient recruitment. AIS, acute ischemic stroke.

Figure 1 depicts the patient selection workflow. A total of 240 patients were enrolled in this study, with a median age of 70 years [interquartile range (IQR): 58.75–78.00] and 143 males (59.6%). The median National Institutes of Health Stroke Scale (NIHSS) score was 8.00 (IQR: 4.00–12.00). Among these, 162 patients (67.5%) comprised the poor prognosis group, characterized by a median age of 72.5 years and median NIHSS score of 11.00 (IQR: 8.00–15.00). Regarding occlusion patterns, 129 patients (53.8%) presented with left-sided infarction. Vascular territories involved included: 70 cases (29.2%) of internal carotid artery (ICA) intracranial segment occlusion; 121 cases (50.4%) of middle cerebral artery (MCA) M1 or proximal M2 segment occlusion; and 49 cases (20.4%) demonstrating combined ICA intracranial segment and MCA M1/proximal M2 occlusions. Treatment modalities consisted of intravenous recombinant tissue plasminogen activator (rt-PA) administration in 57 patients (23.8%) and mechanical thrombectomy (MT) in 32 patients (13.3%).

**Figure 2:**
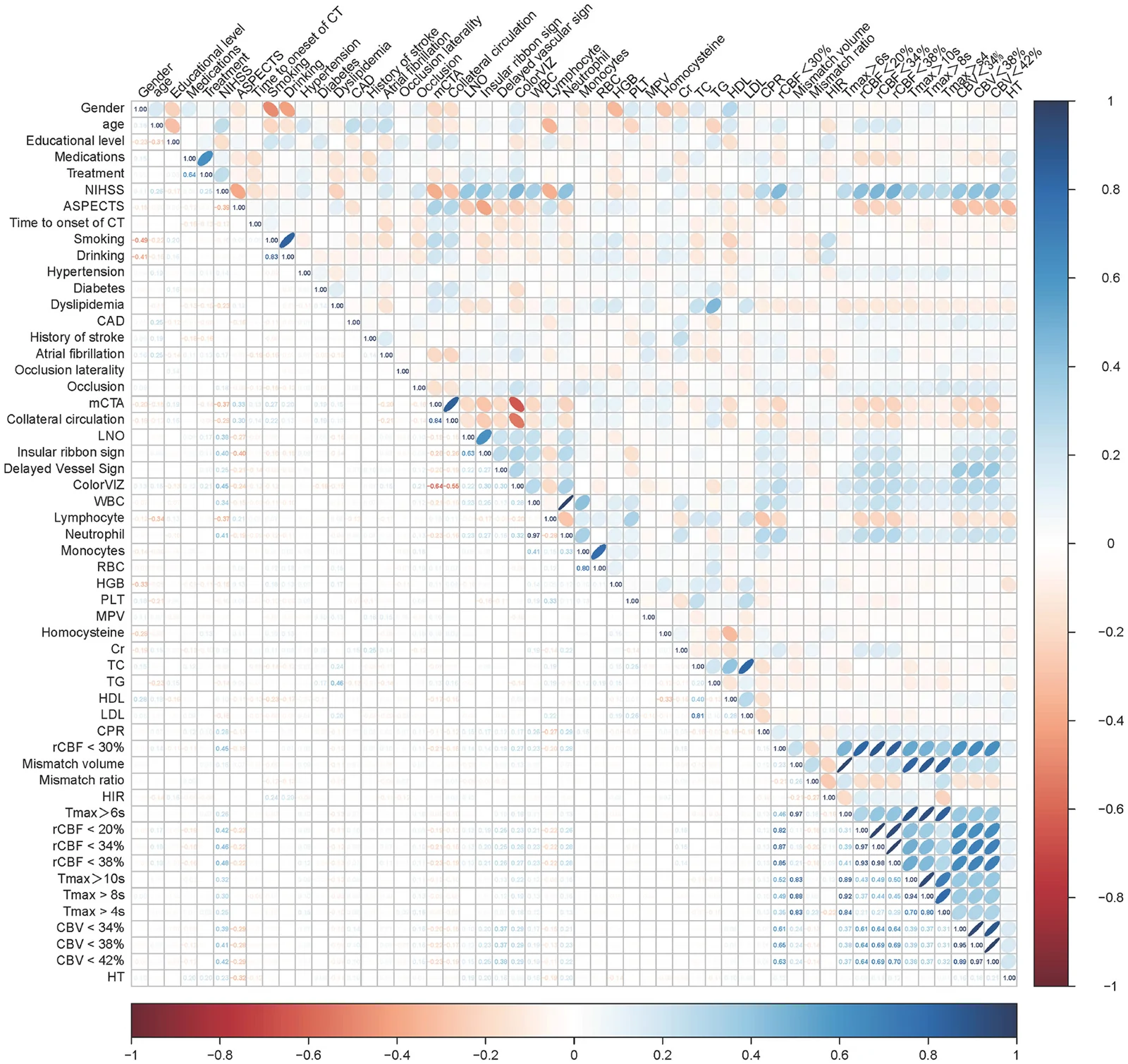
Correlation between demographic, clinical, and neuroimaging data. (NIHSS, National Institutes of Health Stroke Scale; CAD, Coronary Atherosclerotic Heart Disease; mCTA, Multiphase Computed Tomography Angiography; LNO, Lenticular Nucleus Obscuration; WBC, White Blood Cell; RBC, Red Blood Cell; HGB, Hemoglobin; PLT, Platelet; MPV, Mean Platelet Volume; Cr, Creatinine; TC, Total Cholesterol; TG, Triglycerides; HDL, High-density Lipoprotein Cholesterol;LDL, Low-density Lipoprotein Cholesterol; CRP, C-reactive Protein; rCBF, Relative Cerebral Blood Flow; Tmax, Time to maximum of the residue function; CBV, Cerebral Blood Volume; HIR, Hypoperfusion index.)

The cohort was randomly allocated at an 8:2 ratio into a training cohort (*n* = 192) and testing cohort (*n* = 48). Within the training cohort, 130 patients (67.7%) exhibited poor functional outcomes defined as modified Rankin Scale (mRS) ≥ 3. This training dataset served for model development, while the testing cohort was utilized for performance evaluation. Baseline clinical characteristics and radiological features stratified by functional outcome groups are detailed in Table 1. Figure 2 presents the feature selection correlation analysis examining intervariable relationships. A total of 52 variables were incorporated into this study.

**Table 1:** Statistical characteristics of the collected stroke dataset.

Variables	Training cohort			Testing cohort			*p* values
	Poor outcome group (mRS3-6, *n* = 130)	Favorable outcome group (mRS0-2, *n* = 62)	*p* values	Poor outcome group (mRS3-6, *n* = 32)	Favorable outcome group (mRS0-2, *n* = 16)	*p* values	
Demographic characteristics							
Age, M (Q_1_, Q_3_)	72.00 (62.25, 80.00)	61.50 (53.00, 72.50)	<0.001	74.50 (68.00, 79.25)	54.50 (46.75, 72.50)	0.002	0.677
Sex, *n* (%)			0.010			0.676	0.895
Male	69 (53.08)	45 (72.58)		20 (62.50)	9 (56.25)		
Female	61 (46.92)	17 (27.42)		12 (37.50)	7 (43.75)		
Education level, *n* (%)			0.015			0.093	0.679
Illiterate/Primary school	80 (61.54)	26 (41.94)		18 (56.25)	5 (31.25)		
Junior school	24 (18.46)	10 (16.13)		9 (28.13)	3 (18.75)		
High School\ Technical Secondary School	17 (13.08)	17 (27.42)		3 (9.38)	5 (31.25)		
University\ Junior College	9 (6.92)	9 (14.52)		2 (6.25)	3 (18.75)		
Smoking, *n* (%)	25 (19.23)	28 (45.16)	<0.001	11 (34.38)	2 (12.50)	0.207	0.942
Drinking, *n* (%)	20 (15.39)	19 (30.65)	0.014	10 (31.25)	3 (18.75)	0.566	0.308
Medical history, *n* (%)							
Hypertension	81 (62.31)	34 (54.84)	0.323	19 (59.38)	6 (37.50)	0.153	0.326
Diabetes	40 (30.77)	20 (32.26)	0.835	5 (15.63)	6 (37.50)	0.182	0.258
Dyslipidemia	19 (14.62)	19 (30.65)	0.009	7 (21.88)	5 (31.25)	0.724	0.427
Coronary atherosclerotic heart disease	9 (6.92)	8 (12.90)	0.173	6 (18.75)	0 (0.00)	0.165	0.622
Atrial fibrillation	42 (32.31)	11 (17.74)	0.035	8 (25.00)	3 (18.75)	0.903	0.511
History of stroke	16 (12.31)	7 (11.29)	0.839	5 (15.63)	5 (31.25)	0.379	0.111
Clinical characteristics							
NIHSS, M (Q_1_, Q_3_)	11.00 (8.00, 15.00)	3.00 (2.00, 5.00)	<0.001	11.00 (8.75, 15.00)	3.00 (2.75, 5.00)	<0.001	0.952
Treatment, *n* (%)			<0.001			0.276	0.386
Conservative Management	68 (52.31)	51 (82.26)		19 (59.38)	13 (81.25)		
Thrombolysis	41 (31.54)	8 (12.90)		7 (21.88)	1 (6.25)		
Endovascular thrombectomy	21 (16.15)	3 (4.84)		6 (18.75)	2 (12.50)		
Time to onset of CT, hours (IQR)	8.50 (5.00, 13.00)	10.50 (5.00, 17.75)	0.097	8.00 (6.00, 12.50)	11.50 (6.00, 16.00)	0.497	0.444
Hemorrhagic transformation, *n* (%)	22 (16.92)	4 (6.45)	0.047	9 (28.13)	2 (12.5)	0.395	0.108
Laboratory tests							
White Blood Cell count (10^9^/L)	7.93 (6.35, 10.61)	6.67 (5.55, 8.27)	0.002	7.830 (5.37, 9.72)	7.39 (6.22, 8.23)	0.562	0.956
Lymphocyte (10^9^/L)	1.05 (0.78, 1.42)	1.63 (1.09, 1.87)	<0.001	1.17 (0.73, 1.45)	1.75 (1.50, 2.06)	<0.001	0.600
Neutrophil (10^9^/L)	6.05 (4.35, 9.00)	4.23 (3.62, 5.66)	<0.001	6.08 (3.93, 8.05)	4.61 (3.85, 6.02)	0.172	0.984
Platelet count (10^9^/L)	163.00 (127.50, 203.75)	184.00 (141.00, 220.00)	0.044	144.50 (124.50, 181.00)	191.00 (147.75, 224.50)	0.035	0.495
Monocyte count (10^9^/L)	0.45 (0.35, 0.60)	0.40 (0.33, 0.55)	0.158	0.45 (0.31, 0.58)	0.45 (0.38, 0.50)	1.000	0.967
Red Blood Cell count (10^12^/L)	4.81 (4.16, 5.29)	5.03 (4.67, 5.46)	0.026	4.62 (4.12, 5.15)	5.08 (4.13, 5.39)	0.437	0.264
Hemoglobin (g/L)	148.00 (133.00, 163.75)	157.00 (144.00, 168.00)	0.032	146.00 (126.75, 160.25)	157.00 (130.25, 163.50)	0.694	0.324
Mean Platelet Volume (fL)	10.90 (10.10, 11.80)	10.50 (9.80, 11.40)	0.076	10.70 (10.03, 11.13)	10.90 (9.90, 11.63)	0.615	0.300
Homocysteine (μmol/L)	12.90 (9.83, 18.57)	14.45 (11.10, 22.38)	0.135	13.65 (11.00, 21.88)	17.52 (12.59, 18.93)	0.615	0.152
Creatinine (μmol/L)	68.00 (57.00, 82.00)	65.00 (56.50, 79.00)	0.505	69.00 (56.50, 85.00)	62.00 (57.75, 73.25)	0.686	0.873
C-reactive Protein (mg/L)	21.49 (7.85, 45.77)	4.10 (3.10, 14.77)	<0.001	21.90 (4.82, 35.63)	10.46 (5.05, 21.21)	0.457	0.653
Total Cholesterol (mmol/L)	3.80 (3.22, 4.43)	4.00 (3.17, 4.62)	0.438	3.870 (2.94, 4.40)	4.21 (3.79, 4.37)	0.364	0.959
HDL (mmol/L)	0.98 (0.82, 1.20)	0.93 (0.80, 1.03)	0.086	1.03 (0.81, 1.25)	0.90 (0.74, 1.07)	0.165	0.738
LDL (mmol/L)	2.32 (1.04, 2.94)	2.54 (1.92, 3.24)	0.290	2.25 (1.68, 2.89)	2.65 (2.03, 3.04)	0.309	0.603
Triglyceride (mmol/L)	1.21 (0.94, 1.51)	1.37 (1.08, 1.89)	0.017	1.14 (0.90, 1.42)	1.78 (1.06, 2.11)	0.149	0.707
Imaging characteristics							
Occlusion laterality			0.742			0.537	0.698
Left	68 (52.31)	34 (54.84)		17 (53.13)	10 (62.50)		
Right	62 (47.69)	28 (45.16)		15 (46.88)	6 (37.50)		
Occlusion			0.024			0.339	0.648
Supraclinoid ICA occlusion	30 (23.08)	26 (41.94)		8 (25.00)	6 (37.50)		
M1\ M2 occlusion	74 (56.92)	25 (40.32)		14 (43.75)	8 (50.00)		
ICA\ M1\ M2 occlusion	26 (20.00)	11 (17.74)		10 (31.25)	2 (12.50)		
Baseline ASPECTS, M (Q_1_, Q_3_)	8.00 (7.00, 9.00)	9.00 (8.00, 10.00)	<0.001	8.00 (6.00, 9.00)	9.00 (8.00, 9.00)	0.028	0.221
Lenticular nucleus obscuration, *n* (%)	44 (33.85)	3 (4.84)	<0.001	8 (25.00)	3 (18.75)	0.903	0.821
Insular ribbon sign, *n* (%)	50 (38.46)	3 (4.84)	<0.001	10 (31.25)	4 (25.00)	0.911	0.829
Delayed Vessel Sign, *n* (%)	28 (21.54)	5 (8.07)	0.021	8 (25.00)	2 (12.50)	0.530	0.556
ColorViz, *n* (%)			<0.001			0.190	0.424
1	20 (15.39)	32 (51.61)		9 (28.13)	8 (50.00)		
2	52 (40.00)	20 (32.26)		12 (37.50)	6 (37.50)		
3	58 (44.62)	10 (16.13)		11 (34.38)	2 (12.50)		
mCTA, *n* (%)			<0.001			0.270	0.590
1	7 (5.39)	0 (0.00)		2 (6.25)	0 (0.00)		
2	29 (22.31)	5 (8.06)		7 (21.88)	1 (6.25)		
3	49 (37.69)	14 (22.58)		8 (25.00)	7 (43.75)		
4	37 (28.46)	28 (45.16)		10 (31.25)	3 (18.75)		
5	8 (6.15)	15 (24.19)		5 (15.63)	5 (31.25)		
Collateral circulation			<0.001			0.682	1.000
Poor collaterals	85 (65.39)	19 (30.65)		18 (56.25)	8 (50.00)		
Good collaterals	45 (34.62)	43 (69.36)		14 (43.75)	8 (50.00)		
Perfusion imaging parameters							
rCBF <20% (mL), M (Q_1_, Q_3_)	9.68 (1.17, 24.81)	3.30 (0.32, 10.44)	0.004	8.55 (1.26, 20.19)	5.61 (0.43, 12.97)	0.491	0.764
rCBF <30% (mL), M (Q_1_, Q_3_)	17.99 (6.25, 39.36)	6.49 (2.55, 13.59)	<0.001	18.42 (5.53, 30.04)	7.36 (2.30, 28.54)	0.299	1.000
rCBF <34% (mL), M (Q_1_, Q_3_)	19.610 (8.04, 47.62)	7.75 (1.66, 14.41)	<0.001	19.56 (5.17, 40.52)	7.63 (2.69, 30.41)	0.299	0.925
rCBF <38% (mL), M (Q_1_, Q_3_)	30.28 (14.97, 67.33)	10.31 (2.74, 20.18)	<0.001	26.44 (6.24, 59.40)	16.61 (4.13, 43.39)	0.464	0.828
Tmax > 4s (mL), M (Q_1_, Q_3_)	171.65 (77.03, 278.12)	60.00 (26.34, 161.04)	<0.001	151.61 (66.70, 261.58)	74.78 (33.77, 385.99)	0.299	0.721
Tmax > 6s (mL), M (Q_1_, Q_3_)	92.31 (39.52, 156.28)	29.18 (15.06, 66.29)	<0.001	52.79 (34.04, 144.752)	41.72 (13.10, 90.82)	0.412	0.479
Tmax > 8s (mL), M (Q_1_, Q_3_)	70.65 (45.08, 133.32)	25.06 (13.19, 59.04)	<0.001	47.56 (21.50, 158.40)	22.72 (10.36, 99.76)	0.406	0.258
Tmax > 10s (mL), M (Q_1_, Q_3_)	46.76 (23.58, 91.81)	20.62 (7.69, 45.35)	<0.001	37.43 (13.03, 101.46)	34.79 (14.31, 74.73)	0.870	0.725
Mismatch volume (mL), M (Q_1_, Q_3_)	56.64 (18.92, 122.42)	20.28 (7.19, 59.00)	<0.001	36.51 (15.79, 64.20)	28.94 (7.25, 69.11)	0.464	0.422
Mismatch ratio, M (Q_1_, Q_3_)	3.97 (1.91, 13.00)	4.10 (2.36, 9.13)	0.770	3.32 (1.88, 10.81)	3.31 (2.45, 14.80)	0.325	0.959
Hypoperfusion index, M (Q_1_, Q_3_)	0.72 (0.415, 0.958)	0.84 (0.57, 1.05)	0.112	0.79 (0.38, 0.98)	0.76 (0.59, 0.91)	0.718	0.794
CBV <34% (mL)	2.92 (0.77, 13.06)	0.84 (0.03, 3.65)	<0.001	2.50 (0.75, 11.64)	0.85 (0.00, 8.58)	0.180	0.940
CBV <38% (mL)	4.84 (2.04, 17.59)	1.34 (0.05, 4.99)	<0.001	3.89 (1.93, 15.47)	1.47 (0.29, 12.21)	0.250	0.932
CBV <42% (mL)	8.88 (3.24, 22.00)	1.93 (0.15, 5.99)	<0.001	5.62 (2.23, 20.03)	1.81 (1.24, 20.67)	0.279	0.853

NIHSS, National Institutes of Health Stroke Scale; Q_1_, first quartile; Q_3_, third quartile; ASPECTS, Alberta Stroke Program Early Computed Tomography Score; mCTA, Multiphase Computed Tomography Angiography; mCTA scores range from 0 to 5; no patients in this cohort had an mCTA score of 0; HDL,High-density Lipoprotein Cholesterol; LDL, Low-density Lipoprotein Cholesterol; rCBF, Relative Cerebral Blood Flow; Tmax, Time to maximum of the residue function; CBV, Cerebral Blood Volume. ColorViz was graded on a 3-point scale: grade 1 indicated no or minimal collateral delay, grade 2 indicated moderate collateral delay, and grade 3 indicated severe collateral delay or absent collateral visualization.

### Clinical model

Univariate analyses were initially conducted on the training cohort using *t*-tests for continuous variables or chi-square tests for categorical variables to identify clinical features significantly associated with the outcome (*P* < 0.05). Significant variables from univariate analysis were subsequently included in multivariate logistic regression with stepwise selection to identify independent predictors (*P* < 0.05). Baseline comparisons revealed statistically significant associations between poor outcomes and Sex(*P* = 0.011), HGB (*P* = 0.037), WBC count (*P* = 0.001), neutrophil count (*P* < 0.001), lymphocyte count (*P* < 0.001), CRP (*P* < 0.001), age (*P* < 0.001), NIHSS score (*P* < 0.001), occlusion location (*P* = 0.049), atrial fibrillation (*P* = 0.037), alcohol consumption (*P* = 0.016), hyperlipidemia (*P* = 0.011), and treatment modality (*P* < 0.001). Candidate variables with *P* < 0.1 then underwent multivariate logistic regression analysis (Table 2), Time to onset of CT (*P* = 0.015), age (*P* = 0.041), and NIHSS score (*P* < 0.001) as independent predictors of 3-month poor outcomes. These independently significant clinical predictors served as input variables for constructing a clinical prediction model using logistic regression algorithm. The model demonstrated good discriminatory performance, with an area under the curve (AUC) of 0.956 (95% CI: 0.930–0.982) in the training cohort and 0.934 (95% CI: 0.858–1.000) in the testing cohort for predicting 90-day functional outcome.

**Table 2:** Multivariate logistic regression analysis for predicting favorable 90-day functional outcome in patients with anterior circulation large vessel occlusion.

Variable	coefficient	*P* value	OR	95% CI
Age	−0.803	0.041	0.448	(0.207–0.968)
Time to onset of CT, hours	0.724	0.015	2.063	(1.154–3.689)
NIHSS	−5.345	< 0.001	0.005	(0.001–0.045)

In the sensitivity analysis, treatment modality was additionally incorporated into the clinical model. The inclusion of treatment modality did not significantly improve model discrimination. In the training cohort, the AUC increased slightly from 0.956 to 0.958, with no statistically significant difference by the DeLong test (*P* = 0.361). In the testing cohort, the AUC remained unchanged at 0.934, with a DeLong test *P* value of 1.000. These results suggested that the predictive performance of the clinical model was not materially affected by the inclusion of treatment modality (Supplementary Table S1).

### Imaging model

**Figure 3:**
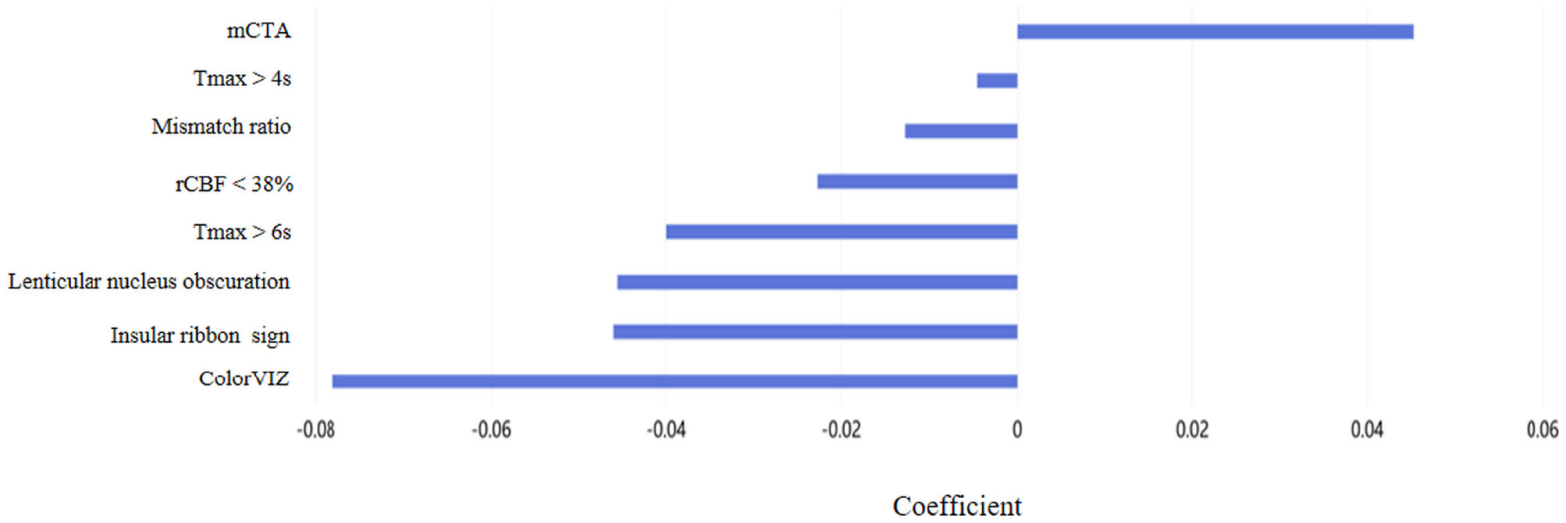
Imaging features and their coefficients used to calculate the imaging score.

For imaging features selection, LASSO regression was employed for dimensionality reduction and feature selection. The optimal regularization parameter (λ) was determined through 10-fold cross-validation, with imaging features corresponding to non-zero coefficients at this λ value retained as final predictors. Eight features demonstrating significant impact on model performance were ultimately preserved (as illustrated in the Figure 3), including: mCTA, Tmax > 4s, Mismatch Ratio, rCBF < 38%, Tmax > 6s, Lenticular Nucleus Obscuration, Insular Ribbon Sign, and ColorViz. These LASSO-selected imaging features served as inputs for constructing an imaging prediction model using logistic regression algorithm. Concurrently, an imaging score was calculated based on the selected features weighted by their respective LASSO regression coefficients. The imaging score computation followed the formula:

### Imaging score = Intercept + Σ(Coefficient × Variable)

Imaging score = 0.322916657 + 0.0452462435 × mCTA + −0.00456425361 × ZTmax>4s + −0.0127362516 × ZMismatch Ratio + −0.0226747319 × ZrCBF < 38% + −0.0399855524 × ZTmax>6s + −0.0455209464 × Lenticular nucleus obscuration + −0.045977015 × Insular ribbon sign+−0.0780020058 × ColorViz.

**Figure 4:**
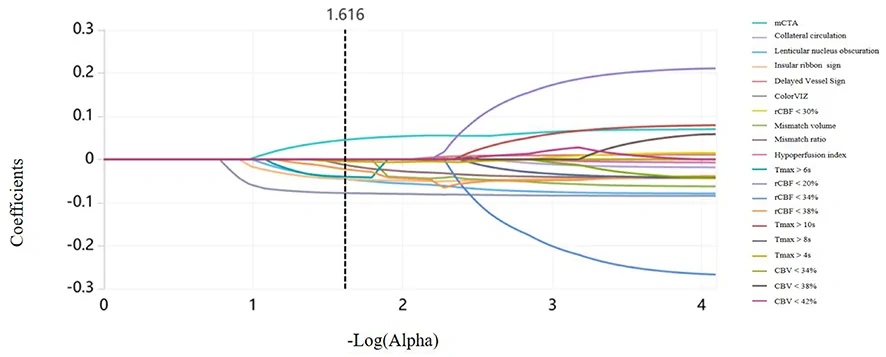
Employing a binary logistic regression model with the Least Absolute Shrinkage and Selection Operator (LASSO) for predictor selection. Each curve in the figure depicts the evolution of individual variable coefficients. The vertical axis represents the coefficient values, the lower horizontal axis corresponds to log(λ), while the upper horizontal axis indicates the count of non-zero coefficients retained in the model at each regularization level.

The model achieved an area under the curve (AUC) of 0.799 (95% CI: 0.734–0.865) for predicting favorable 90-day functional outcome in the training cohort, and an AUC of 0.779 (95% CI: (0.645–0.914) in the testing cohort. Imaging features selected by LASSO and imaging score coefficients are shown in Figure 4.

### Hybrid model

The combined model was a four-variable model incorporating age, NIHSS, onset-to-CT time, and Imaging score. Imaging score represented the weighted composite contribution of the eight imaging variables selected by LASSO. The model was developed in the training cohort and evaluated in the independent testing cohort. Discrimination was assessed using the area under the receiver operating characteristic curve with 95% confidence intervals. The hybrid model achieved AUCs of 0.956 (95% CI: 0.931–0.981) and 0.941 (95% CI: 0.871–1.000) in the training and testing cohorts, respectively, for predicting favorable 90-day functional outcome.

**Figure 5:**
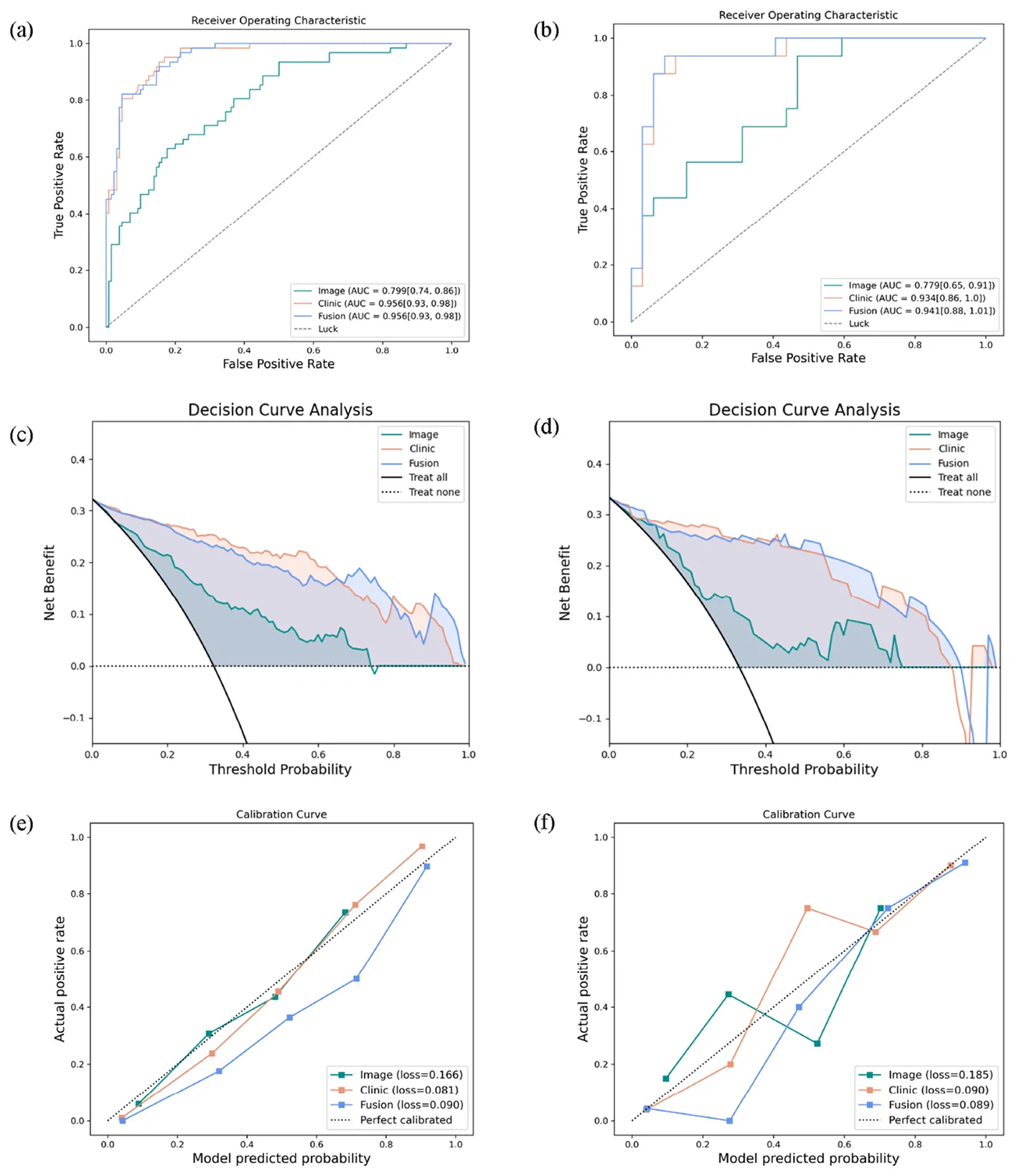
ROC curve, calibration curve, and clinical decision curve. **(a)** ROC curve of the training cohort; **(b)** ROC curve of the testing cohort; **(c)** the training cohort decision curve; **(d)** testing cohort decision curve; **(e)** Calibration curve of the training cohort; **(f)** Calibration curve of the testing cohort.

All models were evaluated in the training and held-out testing cohorts using the AUC, accuracy, sensitivity, and specificity (Table 3). In the training cohort, the clinical, imaging, and hybrid models achieved AUCs of 0.956, 0.799, and 0.956, respectively (Figures 5a, b). The corresponding AUCs in the testing cohort were 0.934 (95% CI, 0.858–1.0000), 0.779 (95% CI, 0.645–0.914), and 0.941 (95% CI, 0.871–1.0000), respectively. Paired DeLong testing (Table 4) showed no significant difference between the clinical and hybrid models in either the training cohort (ΔAUC = −0.0001; 95% CI, −0.0079 to 0.0080; *P* = 0.988) or the testing cohort (ΔAUC = 0.0078; 95% CI, −0.0071 to 0.0227; *P* = 0.305). Calibration plots showed generally acceptable agreement between predicted and observed probabilities in the training cohort, whereas greater variability was observed in the testing cohort because of its limited sample size (Figures 5e, f). Decision curve analysis showed broadly comparable net-benefit profiles for the clinical and hybrid models across the evaluated threshold range (Figures 5c, d). The hybrid model did not show improved optimism-corrected discrimination compared with the clinical model (Supplementary Table S2). In repeated stratified 5-fold cross-validation performed 20 times, the mean AUCs were 0.942 (SD, 0.0026) for the clinical model, 0.747 (SD, 0.0108) for the imaging model, and 0.939 (SD, 0.0038) for the hybrid model, indicating no measurable incremental improvement in discrimination after adding the imaging score (Supplementary Table S3).

**Table 3:** Predictive performance of the models for 90-day functional outcome in AIS-LVO.

Model	AUC (95% CI)	Accuracy	Sensitivity	Specificity
Training cohort				
Clinical model	0.956 (0.930–0.982)	0.885	0.807	0.923
Imaging model	0.799 (0.734–0.865)	0.734	0.677	0.762
Hybrid model	0.956 (0.931–0.981)	0.865	0.919	0.839
Testing cohort				
Clinical model	0.934 (0.858–1.000)	0.896	0.813	0.937
Imaging model	0.779 (0.645–0.914)	0.646	0.563	0.688
Hybrid model	0.941 (0.871–1.000)	0.917	0.875	0.938

AUC area under the receiver operating characteristic curve,CI confidence interval.

**Table 4:** Paired DeLong comparison of the clinical and hybrid models.

Cohort	Clinical AUC	Hybrid AUC	ΔAUC (95% CI)	Z	*P* value
Training cohort	0.956	0.956	−0.0001 (−0.0079 to 0.0080)	0.015	0.988
Testing cohort	0.934	0.941	+0.0078 (−0.0071 to 0.0227)	1.026	0.305

Delta AUC was defined as hybrid-model AUC minus clinical-model AUC. *P* values were obtained using the two-sided paired DeLong test. AUC, area under the receiver operating characteristic curve; CI, confidence interval.

**Figure 6:**
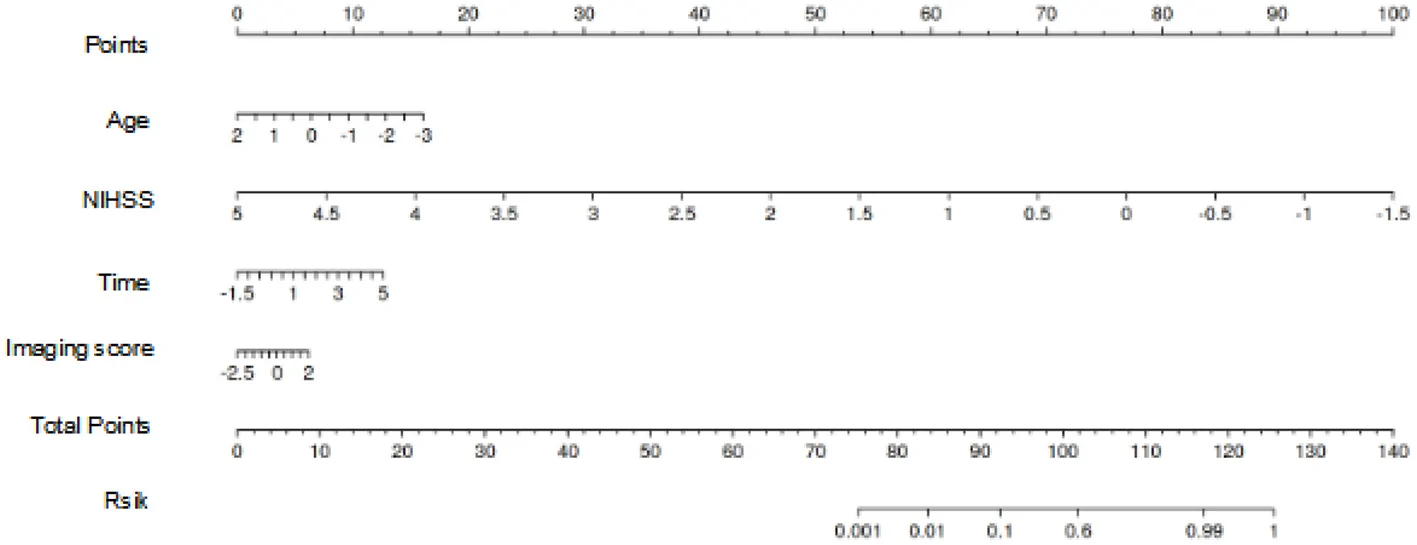
Nomogram for predicting 90-day functional outcome in patients with AIS-LVO. This nomogram integrates four indicators—age, NIHSS score, time from onset to first CT scan, and imaging score—to predict the risk of poor outcomes in patients with AIS. Imaging score was a weighted composite of the eight imaging variables selected by LASSO.

To enhance clinical applicability and interpretability, a nomogram incorporating the imaging score from the imaging model alongside independently significant clinical predictors was developed. This visual tool quantitatively displays the contribution weights of individual predictors, facilitating personalized risk estimation and clinical decision-making (Figure 6). Each variable corresponds to a score ranging from 0 to 100, with the total score derived from summing the scores of four variables. The estimated probability of poor outcome is obtained by projecting the total score onto the nomogram scale. These findings underscore the robust clinical validity of the nomogram in both training and validation cohorts.

### SHAP analysis

**Figure 7:**
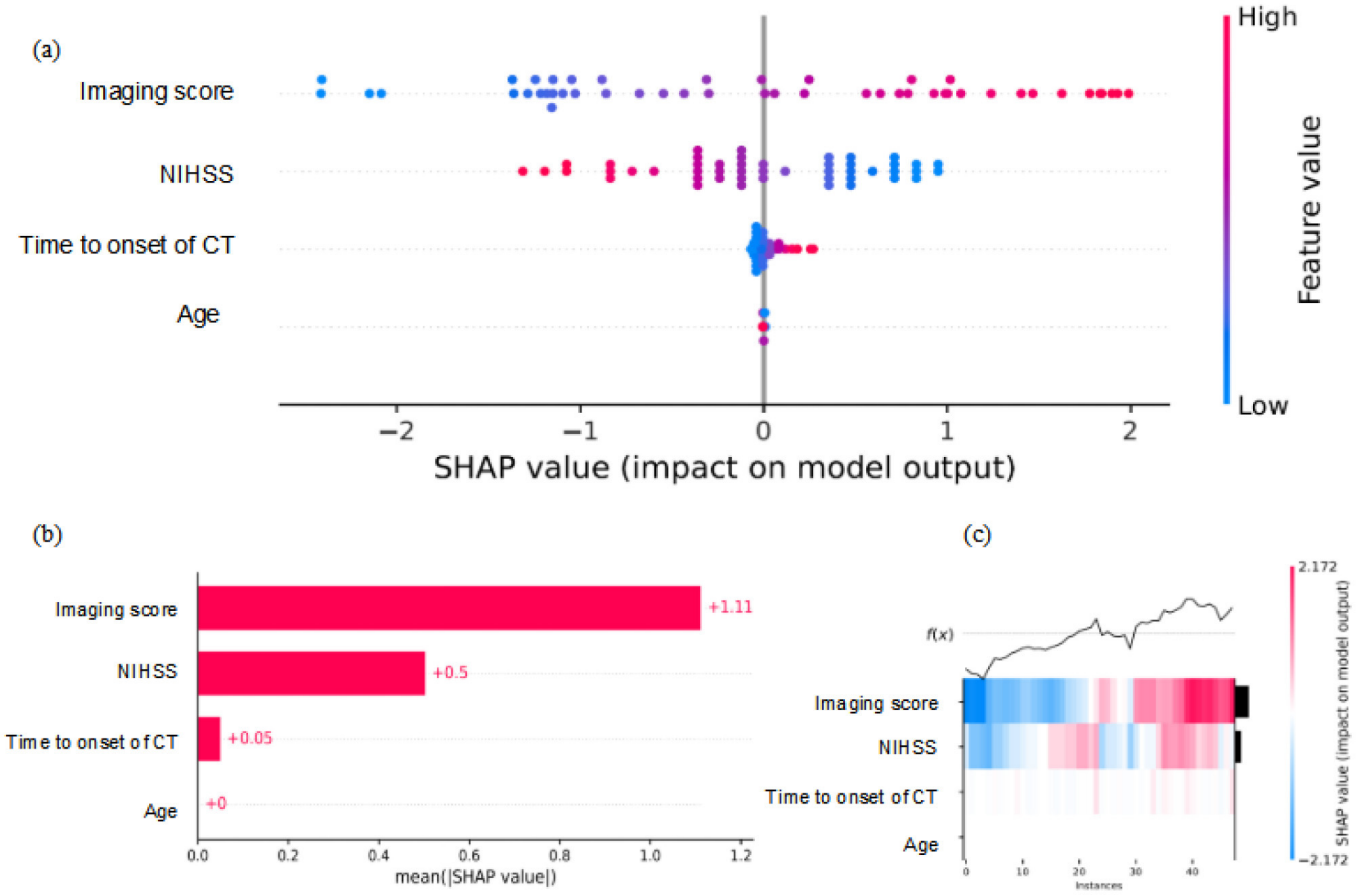
SHAP-based interpretation of the clinical-imaging model for predicting favorable 90-day functional outcome. **(a)** SHAP summary plot showing the distribution and direction of each predictor's contribution to model output. Positive SHAP values indicate an increased predicted probability of favorable 90-day functional outcome, whereas negative SHAP values indicate a decreased probability. The color gradient represents feature values from low to high. **(b)** Global feature importance ranked by the mean absolute SHAP value. Imaging score showed the largest contribution to model prediction, followed by NIHSS score, onset-to-CT time, and age. **(c)** SHAP heatmap illustrating the contribution patterns of individual predictors across patient-level predictions.

SHapley Additive Explanations (SHAP) were used to interpret the hybrid model incorporating age, NIHSS, onset-to-CT time, and the Imaging score. SHAP values were calculated on the logistic model-output, or log-odds, scale. Global feature importance was quantified using the mean absolute SHAP value across all evaluated patients. A SHAP summary plot was generated to display both the magnitude and direction of each predictor's contribution to the model output. Each point represents an individual patient, and its horizontal position indicates the corresponding SHAP value. Positive SHAP values increased the log-odds of favorable 90-day functional outcome, whereas negative SHAP values decreased them. Points located farther from zero represent a greater influence on the prediction. Feature values are color-coded, with red indicating higher values and blue indicating lower values, thereby illustrating how variations in each predictor affect model predictions. Because the composite imaging score was derived from eight LASSO-selected imaging variables, its SHAP value represents the aggregated contribution of these imaging characteristics rather than the importance of any single imaging variable. In this analysis, the key determinants ranked by importance were imaging score, elevated admission NIHSS score, time from stroke onset to initial CT scanning, and age (Figure 7).

SHAP analysis showed that the imaging score had the largest mean absolute SHAP value among the four model inputs, indicating the greatest overall contribution to predictions within the combined model. Higher imaging score values were generally associated with increased model output for favorable functional outcome. Admission NIHSS ranked second in global importance and showed an inverse relationship with prognosis, with higher NIHSS values contributing to a lower predicted likelihood of favorable outcome. These findings suggest that the composite imaging score and baseline neurological severity were the principal contributors to predictions generated by the four-variable model. However, the SHAP importance of the imaging score reflects its contribution within the fitted model and should not, by itself, be interpreted as evidence of independent or incremental predictive value beyond the clinical variables.

## Discussion

This study developed and validated an interpretable hybrid model that integrates three routine clinical predictors (age, admission NIHSS score, and onset-to-CT time) with a composite multimodal CT-derived imaging score to estimate 90-day functional outcome in patients with anterior-circulation AIS-LVO. The clinical and hybrid models both achieved high discrimination in the independent testing cohort (AUC 0.934 and 0.941), while the imaging-only model performed more modestly. Although addition of the imaging score produced no statistically significant improvement in AUC, SHAP analysis ranked the imaging score as the dominant contributor to individual predictions, indicating that collateral and perfusion information supplies complementary, patient-level insight rather than merely incremental global discrimination.

Admission NIHSS score integrates the functional consequences of lesion location, eloquent-tissue involvement, ischemic burden, and collateral failure, while age captures differences in vascular reserve, comorbidity, and recovery capacity. These variables have consistently ranked among the strongest determinants in clinical and multimodal stroke outcome models (7, 14, 21). In the present cohort, the marked separation in NIHSS score and age between favorable- and unfavorable-outcome groups probably contributed to the AUC above 0.93. The lower discrimination of the imaging-only model in the test set further indicates that quantitative imaging should complement, rather than replace, standardized neurological examination. Conversely, the high prognostic value of NIHSS should not be interpreted as making vascular or tissue imaging dispensable, because clinical scales alone do not reliably characterize occlusion status or residual tissue viability (22). Clinical manifestations provide only a transient reflection of neurological deficits. Some penumbral tissue may remain functionally silent yet physiologically viable, with metabolic injury not having progressed to irreversible cell death, thereby obscuring the true extent of neurological impairment. In contrast, the imaging score—constructed using a relative cerebral blood flow threshold of < 38%, time-to-maximum (Tmax) delay, and ColorViz-quantified collateral hemodynamics—more effectively reveals the underlying physiological mechanisms and compensatory reserve of ischemic brain tissue. Onset-to-CT time requires particularly careful interpretation. After multivariable adjustment, a longer interval was associated with a greater probability of favorable outcome, a direction that is inconsistent with the causal effect expected from ongoing untreated ischemia. The most credible explanation is a selection and triage effect within this retrospective imaging cohort: patients reaching multimodal CT at later times are likely to be enriched for slower infarct progression, preserved collaterals, smaller established cores, or uncertainty in the recorded last-known-well time, whereas very early presentation may be enriched for abrupt and clinically severe strokes. Suppression effects may also arise because onset-to-CT time is correlated with baseline severity, treatment selection, referral pathway, and imaging phenotype. Late-window trials demonstrate benefit when delayed patients are selected by favorable tissue or collateral profiles, within systems that continue to minimize avoidable delay (8, 9, 23). Future studies should distinguish onset-to-door, door-to-CT, interhospital transfer time, last-known-well uncertainty, and onset-to-reperfusion time, and should test their interactions with collateral status and infarct-core burden.

The imaging score captured three pathophysiologically linked domains. Lenticular nucleus obscuration and the insular ribbon sign reflect early parenchymal injury on non-contrast CT; rCBF < 38%, Tmax > 4 s, Tmax > 6 s, and mismatch ratio describe the extent and severity of perfusion disturbance; and mCTA and ColorViz summarize the adequacy and temporal efficiency of collateral filling. Their joint selection supports a model in which outcome is determined by the interaction between established tissue injury, threatened but potentially salvageable tissue, and the hemodynamic reserve that sustains it. This interpretation is consistent with the established value of automated perfusion thresholds, multiphase CTA, and time-resolved collateral assessment in stroke triage and outcome estimation (11, 13, 16–18, 24). These findings align with and extend recent literature. Clinical severity and age remain the dominant predictors across multiple cohorts of LVO stroke, whether modeled by logistic regression or more complex machine-learning algorithms (14, 25). Multiphase CTA collateral grading and ColorViz temporal maps have repeatedly been shown to correlate with final infarct volume and functional recovery (11, 15, 16), and CTP-derived Tmax and rCBF thresholds are central to late-window selection criteria established by the DAWN and DEFUSE 3 trials (8, 9). Prior imaging-only or radiomics models typically report AUCs in the 0.70–0.85 range, comparable to our imaging score, whereas hybrid clinical–imaging models frequently reach 0.85–0.95 (21). In the present work, we used the least absolute shrinkage and selection operator (LASSO) on both conventional and advanced CT features to build a simple, interpretable imaging-derived score. This approach may offer a comparatively interpretable option, as opposed to the high-dimensional, hard-to-decode feature outputs obtained from deep-learning models. The lack of incremental AUC following the addition of the imaging score may be attributed to the following reasons. First, the clinical model already exhibited relatively strong discriminative performance, which leaves only modest potential for further improvement. Second, NIHSS partially encodes the downstream functional expression of the same ischemic burden and collateral failure measured by CT, creating substantial informational redundancy. Clinically, this nomogram serves as a readily-available decision-support tool. In resource-limited or rural settings where comprehensive multimodal post-processing imaging may be delayed, favorable risk stratification can be achieved using the clinical triad alone. When CTP and ColorViz are accessible, the imaging score further optimizes individualized risk assessment and identifies patients whose collateral or perfusion status is discordant with clinical presentation. Such refined assessment facilitates shared clinical decision-making regarding aggressive endovascular therapy, or intensified secondary prevention strategies (26, 27). The observed performance lies within the range reported by contemporary stroke prognostic models, although direct numerical comparisons are limited by differences in case mix, outcome definition, treatment exposure, feature timing, and validation strategy. Heo et al. (21) reported an AUROC of 0.888 in a large clinical dataset, Brugnara et al. (14) demonstrated the value of multimodal clinical and imaging information after endovascular treatment, and Ozkara et al. (28) reported an AUROC of 0.958 with LightGBM in a smaller proximal-MCA cohort. Subsequent imaging-focused and interpretable hybrid studies have similarly emphasized the complementary roles of quantitative imaging and transparent model explanation (7, 20). Recent advances in artificial intelligence have further highlighted the value of integrating complementary imaging information in acute ischemic stroke. Li et al. (29) developed a deep feature-fusion framework that combines information from multiple CT perfusion parameter maps for ischemic lesion segmentation, demonstrating the potential of cross-parametric hemodynamic information to improve lesion characterization. Similarly, Ma et al. (30) proposed LVONet, an automated approach that exploits interhemispheric vascular differences for the classification of large-vessel occlusion. These studies illustrate the increasing role of multimodal and spatially complementary imaging features in stroke assessment. However, their primary objectives were lesion segmentation and occlusion classification rather than long-term functional prognosis. Unlike prior studies, the present study integrates routine clinical predictors together with NCCT, multiphase CTA, ColorViz maps and CTP into the emergency workflow. By condensing eight correlated imaging variables into a single clinically interpretable score, we compared the predictive performance of clinical-only, imaging-only and hybrid models. We aimed to investigate the prognostic value of this multi-modal-CT-derived score for acute ischemic stroke and assess whether imaging features could add discriminative value over clinical variables, providing preliminary evidence for multi-modal CT implementation in emergency stroke care. Of note, this imaging score did not yield significant improvement in overall discrimination relative to the clinical model. Nevertheless, it offers supplementary physiological information about collaterals, hypoperfusion severity and brain tissue status, which cannot be adequately captured by clinical severity assessment alone. Although treatment modality (mechanical thrombectomy or intravenous thrombolysis) showed a significant univariate association with outcome, it was not retained as an independent predictor in the final multivariable model. Sensitivity analysis confirmed that adding treatment modality did not materially improve model discrimination (*P* = 0.361). This finding indicates that the pretreatment physiological state—captured by the imaging score and NIHSS—largely determines the patient's prognostic trajectory. In large vessel occlusion, the threshold for futile recanalization appears preset by baseline brain tissue status: when the infarct core is excessively large or collateral compensation is severely exhausted, even technically successful thrombectomy may fail to translate into meaningful functional recovery (23, 31). Acute anterior circulation ischemic stroke comprises heterogeneous subtypes, risk factors, and clinical characteristics that profoundly influence prognosis. Therefore, precise radiological interpretation, rigorous collateral circulation assessment, and the individualized integration of intravenous thrombolysis and mechanical thrombectomy are essential for optimizing clinical outcomes.

## Limitations

This study has several limitations. First, this was a single-center retrospective study with a relatively modest sample size, which may limit the generalizability of the findings. Although bootstrap internal validation revealed that the model maintained good discriminative performance after optimism correction, the relatively limited sample size and number of candidate predictors may still pose a risk of overfitting. Hence, external validation based on large-sample multicenter prospective cohorts is required to further verify the robustness and generalizability of the model. Second, the staged modeling strategy was adopted to improve clinical interpretability and facilitate nomogram construction; however, stepwise variable selection and post-selection model fitting may introduce variable-selection instability and optimistic performance estimates. Unified penalized approaches, such as LASSO or elastic net regression incorporating all candidate clinical and imaging predictors simultaneously, should be further compared in future studies. Third, the retrospective design may have introduced selection bias, information bias, and residual confounding. In particular, treatment modality was associated with 90-day outcome in univariate analysis but was not retained in the final multivariable model. Because treatment decisions may be influenced by baseline stroke severity, imaging findings, onset-to-imaging time, and physician judgment, treatment-related confounding cannot be completely excluded. Finally, the follow-up period was limited to 90 days, which precluded assessment of longer-term functional trajectories. Future studies should incorporate external validation, dynamic model updating, and extended follow-up to improve the clinical applicability of the proposed model.

## Conclusion

This study developed an interpretable prognostic model for predicting 90-day functional outcomes in patients with acute ischemic stroke and anterior-circulation large-vessel occlusion. Age, admission NIHSS score, and onset-to-CT time were identified as key clinical predictors, while eight multimodal CT-derived imaging features were integrated into a composite imaging score. The resulting hybrid model incorporated these three clinical predictors together with the imaging score and demonstrated good discrimination in both the training and testing cohorts. Although the addition of the imaging score did not significantly improve overall discrimination compared with the clinical model alone, it provided complementary information on collateral circulation, cerebral perfusion, and early ischemic changes. These findings support the potential value of integrating routinely available clinical characteristics with multimodal CT-derived information for individualized prognostic assessment in patients with AIS-LVO. Further external validation in larger, multicenter prospective cohorts is warranted before clinical implementation.

## Data Availability

The original contributions presented in the study are included in the article/Supplementary material, further inquiries can be directed to the corresponding author.
