# Supplementary material for: An interpretable machine learning model for predicting prognosis in acute ischemic stroke with large vessel occlusion
Source: Front Neurol. 2026 Sep 9;17:1842324. doi: 10.3389/fneur.2026.1842324 (PMC13597449; doi:10.3389/fneur.2026.1842324)
Supplement: Supplementary file 1 [file Supplementary_file_1.docx]

**Detailed Guide for the imaging score Developed for This Study**

1. Background

The imaging score was developed specifically for the present study to predict functional outcome in patients with acute ischemic stroke with Large Vessel Occlusion by integrating multi-modal imaging features. The imaging score was calculated based on selected features weighted by their respective LASSO regression coefficients, and the detailed computation method, variable definitions, and coefficient derivation are provided below to ensure reproducibility.

2. Definition of Variables in the Formula

All variables included in the imaging score were derived from multi-modal imaging (CT angiography and perfusion CT) or imaging signs, with detailed definitions as follows:

a. mCTA: Score of multiphase CT angiography (mCTA) for collateral circulation, utilizing the 0–5 point scale proposed by Bijoy K. Menon et al. (see reference in the main manuscript), which is consistent with the mCTA collateral grading standard described in the main manuscript.Tmax>4s: Volume of tissue with time to maximum of the residue function (Tmax) > 4 seconds, measured via automated intelligent post-processing of perfusion CT (CTP) images using the AI-powered imaging analytics software uAI Research Portal (Shanghai United Imaging Intelligence)(Wu et al., 2023).
b. Mismatch volume and mismatch ratio: The ischemic core was estimated as the volume of tissue with rCBF < 30%, whereas the hypoperfused region was defined as the volume of tissue with Tmax > 6 s. Mismatch volume was calculated as the Tmax > 6 s volume minus the rCBF < 30% volume. Mismatch ratio was calculated as the Tmax > 6 s volume divided by the rCBF < 30% volume. These parameters quantify the extent of hypoperfused tissue relative to the estimated ischemic core.
c. rCBF<38%: Volume of tissue with relative cerebral blood flow (rCBF) < 38%, reflecting severe cerebral hypoperfusion. Additional rCBF thresholds (rCBF ＜20%,rCBF<30%, rCBF<34%, rCBF <38%) were extracted in this study, and rCBF<38% was selected as the variable in the imaging score via LASSO regression. All rCBF parameters were measured from CTP images post-processed by uAI Research Portal [33].
d. Tmax>6s: Volume of tissue with Tmax > 6 seconds, measured via CTP post-processing using uAI Research Portal [33], indicating prolonged hypoperfusion. This parameter is also used for calculating the Hypoperfusion Index (HIR).
e. Lenticular nucleus obscuration: Binary variable (0 = absent, 1 = present), defined as obscuration of the lenticular nucleus on non-contrast CT, which is a sign of early ischemic change.
f. Insular ribbon sign: Binary variable (0 = absent, 1 = present), defined as loss of the insular ribbon on non-contrast CT, indicating ischemia in the middle cerebral artery (MCA) territory.
g. ColorViz: Score of collateral circulation assessed by ColorViz fusion images (FastStroke, GE Healthcare), based on a dual-parameter collateral circulation scoring system using temporally resolved color maps. This system employed axial color-coded maximum intensity projections (MIPs) to categorize both collateral filling extent and delay into three tiers each. Collateral extent was graded as follows: Normal/Near-normal (>90%): Vascular territories ipsilateral to the occlusion demonstrated near-complete symmetry with the contralateral hemisphere; Moderate reduction (50–90%): Visualized vasculature exhibited 10–50% reduced coverage compared with the contralateral side; Marked reduction (<50%): Vascular territories encompassed <50% of the contralateral extent. Collateral delay was graded as follows: No delay: Predominantly red coloration within the occluded territory, indicating synchronous opacification; Single-phase delay: Green predominance, reflecting a one-phase lag relative to contralateral circulation; Two-phase delay/Absent collaterals: Blue appearance or complete absence of visualization, indicating ≥2-phase delay. ColorViz is scored on a 1–3 scale: 1 indicates normal or near-normal collateral filling with no obvious delay, mainly shown in red; 2 indicates moderate collateral delay, usually a one-phase delay, mainly shown in green; and 3 indicates severe collateral delay or absent collateral visualization, corresponding to a delay of two or more phases or no visible collateral vessels, mainly shown in blue or no visualization. Lower scores indicate better collateral circulation, whereas higher scores indicate poorer collateral status.
h. Additional Extracted CTP Parameters: Other extracted parameters included rCBF<32%, rCBF<34%, Tmax>8s, Tmax>10s, cerebral blood volume (CBV)<34%, CBV<38%, CBV<42%, and Hypoperfusion Index (HIR), all derived from CTP images post-processed by uAI Research Portal (Wu et al., 2023).
i. Hypoperfusion Index (HIR): Defined as the volumetric ratio of tissue with Tmax>10s to tissue with Tmax>6s.

4. Imaging score Calculation Steps

To ensure reproducibility, the step-by-step calculation process is provided as follows:

1) Collect all variables defined above from each study participant’s multi-modal imaging data, and assign values according to the variable definitions (e.g., binary variables: 0 or 1; continuous variables: measured volume or ratio).
2) Multiply each variable by its corresponding LASSO regression coefficient (as shown in the formula).
3) Sum all the products obtained in Step 2, and add the intercept (0.322916657) to obtain the final imaging score.

3. Imaging score Calculation Formula

The imaging score computation followed the formula:

Imaging score = 0.322916657＋0.0452462435×mCTA＋-0.00456425361×ZTmax>4s ＋-0.0127362516 ×ZMismatch Ratio ＋ -0.0226747319 × ZrCBF<38% ＋ -0.0399855524×ZTmax>6s ＋ -0.0455209464 ×Lenticular nucleus obscuration ＋ -0.045977015×Insular ribbon sign＋-0.0780020058×ColorViz

The continuous variables—Tmax > 4 s, mismatch ratio, rCBF < 38%, and Tmax > 6 s—were entered into the imaging score formula as z-score standardized values rather than raw measurements. Standardization was performed using the mean and standard deviation derived exclusively from the training cohort (n = 192), according to the following equation:

Z = (X − μtraining) / σtraining

Specifically, the standardized variables were calculated as follows:

ZTmax>4s = ([Tmax > 4 s] − 190.214) / 188.022

ZMismatch ratio = (Mismatch ratio − 13.933) / 32.986

ZrCBF<38% = ([rCBF < 38%] − 43.194) / 63.900

ZTmax>6s = ([Tmax > 6 s] − 113.897) / 154.205

The categorical and ordinal imaging variables were coded as follows. The mCTA collateral score was entered directly as an ordinal variable ranging from 0 to 5. ColorViz was entered directly as an ordinal variable ranging from 1 to 3: 1. Lenticular nucleus obscuration and the insular ribbon sign were coded as binary variables, with 0 indicating absence and 1 indicating presence.

**Summary of standardization parameters and coding schemes**

| **Variable** | **Type** | **Coding or transformation** | **Training mean** | **Training SD** |
| --- | --- | --- | --- | --- |
| Tmax > 4 s | Continuous | Z = (X − 190.214) / 188.022 | 190.214 | 188.022 |
| Mismatch ratio | Continuous | Z = (X − 13.933) / 32.986 | 13.933 | 32.986 |
| rCBF < 38% | Continuous | Z = (X − 43.194) / 63.900 | 43.194 | 63.900 |
| Tmax > 6 s | Continuous | Z = (X − 113.897) / 154.205 | 113.897 | 154.205 |
| mCTA | Ordinal | Original score entered directly: 0–5 | — | — |
| ColorViz | Ordinal | 1 = normal/near-normal; 2 = moderate delay; 3 = severe delay or absent collaterals | — | — |
| Lenticular nucleus obscuration | Binary | 0 = absent; 1 = present | — | — |
| Insular ribbon sign | Binary | 0 = absent; 1 = present | — | — |

4. Derivation of LASSO Regression Coefficients

The coefficients and intercept in the imaging score formula were derived from LASSO regression analysis performed on the study’s training cohort, aiming to select the most predictive features and avoid overfitting. The detailed process is described as follows:

1. Initial feature pool: 20 candidate features including imaging signs, perfusion parameters, and mCTA collateral scores were initially included.
2. LASSO regression settings: Penalty parameter λ was selected via 10-fold cross-validation, minimizing the mean squared error; features with non-zero coefficients were retained.
3. Final feature selection: The 8 variables included in the imaging score were the features with non-zero coefficients after LASSO regression, and their respective coefficients were used for weighted calculation.
4. Applicability Notes: The imaging score was developed for patients with acute ischemic stroke who underwent multi-modal CT, including non-contrast CT, mCTA, and perfusion CT. It is not applicable to other populations (e.g., hemorrhagic stroke, transient ischemic attack) or patients without complete multi-modal imaging data.

5.To evaluate whether treatment modality influenced model performance, treatment modality was additionally incorporated into the clinical model as a sensitivity analysis. Model discrimination was compared using the DeLong test in both the training and testing cohorts.

Supplementary Table S1

| Model | Training AUC | DeLong P value | Testing AUC | DeLong P value |
| --- | --- | --- | --- | --- |
| Original clinical model | 0.956 | — | 0.934 | — |
| Clinical model + treatment modality | 0.958 | 0.361 | 0.934 | 1.000 |

6.To assess potential model overfitting and internal stability, EPV and bootstrap internal validation were evaluated for the clinical, imaging, and hybrid models. Bootstrap internal validation was performed with 1000 resamples, and optimism-corrected AUCs with 95% confidence intervals were reported.

**Supplementary Table S2**

| **Cohort** | **Model** | **Observed AUC** | **Bootstrap mean AUC** | **Bootstrap SD** | **95% bootstrap percentile interval** | **Resamples** |
| --- | --- | --- | --- | --- | --- | --- |
| Training | Imaging | 0.799 | 0.797 | 0.0339 | 0.728–0.858 | 1,000 |
| Training | Clinical | 0.956 | 0.956 | 0.0131 | 0.927–0.979 | 1,000 |
| Training | Hybrid | 0.956 | 0.956 | 0.0127 | 0.927–0.978 | 1,000 |
| Testing | Imaging | 0.779 | 0.776 | 0.0718 | 0.624–0.906 | 1,000 |
| Testing | Clinical | 0.934 | 0.931 | 0.0394 | 0.842–0.996 | 1,000 |
| Testing | Hybrid | 0.941 | 0.939 | 0.0370 | 0.858–0.998 | 1,000 |

*The 95% bootstrap percentile interval was defined by the 2.5th and 97.5th percentiles of the bootstrap AUC distribution. AUC, area under the receiver operating characteristic curve; SD, standard deviation.*

7.Repeated cross-validation results

Supplementary Table S3

| **Model** | **Repetitions** | **Mean AUC** | **SD** | **Median AUC** | **2.5th percentile** | **97.5th percentile** |
| --- | --- | --- | --- | --- | --- | --- |
| Clinical | 20 | 0.942 | 0.0026 | 0.943 | 0.938 | 0.946 |
| Imaging | 20 | 0.747 | 0.0108 | 0.747 | 0.730 | 0.763 |
| Hybrid | 20 | 0.939 | 0.0038 | 0.949 | 0.932 | 0.945 |
